# Calcium carbide–induced derangement of hematopoiesis and organ toxicity ameliorated by cyanocobalamin in a mouse model

**DOI:** 10.1186/s42826-022-00136-1

**Published:** 2022-08-12

**Authors:** Pherah A. Ouma, Victoria K. Mwaeni, Peris W. Amwayi, Alfred Orina Isaac, James Nyabuga Nyariki

**Affiliations:** 1 Department of Biochemistry and Biotechnology, Technical University of Kenya, P. O. Box 52428, 00200 Nairobi, Kenya; 2 Department of Pharmaceutical Sciences and Technology, School of Health Sciences and Technology, Technical University of Kenya, P. O. Box 52428, 00200 Nairobi, Kenya

**Keywords:** Calcium carbide, Vitamin B12, Hematopoiesis, Toxicity

## Abstract

**Background:**

Calcium carbide (CaC_2_) is a chemical primarily used in the production of acetylene gas. The misuse of CaC_2_ to induce fruit ripening is a global challenge with a potential adverse effects to human health. Additionally, CaC_2_ is known to contain some reasonable amount of arsenic and phosphorous compounds that are toxic and pose a danger to human health when ingested. The current study sought to characterize CaC_2_ toxicity and elucidate any protective effects by cyanocobalamin (vitamin B_12_), a well-established antioxidant and anti-inflammatory bio-molecule. Female Swiss white mice were randomly assigned into three groups; the first group was the control, while the second group was administered with CaC_2_. The third group received CaC_2_ followed by administration of vitamin B12. The mice were sacrificed at 60 days post treatment, hematological, biochemical, glutathione assay, cytokine ELISA and standard histopathology was performed.

**Results:**

CaC_2_ administration did not significantly alter the mice body weight. CaC_2_ administration resulted in a significant decrease in packed cell volume (PCV), hemoglobin (Hb), red blood cells (RBCs) and RBC indices; indicative of CaC_2_-driven normochromic microcytic anaemia. Further analysis showed CaC_2_-driven leukopenia. Evidently, vitamin B_12_ blocked CaC_2_-driven suppression of PCV, Hb, RBCs and WBCs. Monocytes and neutrophils were significantly up-regulated by CaC_2_. CaC_2_-induced elevation of aspartate aminotransferase (AST), alanine aminotransferase (ALT) and bilirubin signaled significant liver damage. Notably, vitamin B_12_ stabilized AST, ALT and bilirubin in the presence of CaC_2,_ an indication of a protective effect. Histopathological analysis depicted that vitamin B_12_ ameliorated CaC_2_-driven liver and kidney injury. CaC_2_ resulted in the depletion of glutathione (GSH) levels in the liver; while in the brain, kidney and lungs, the GSH levels were elevated. CaC_2_ administration resulted in elevation of pro-inflammatory cytokines TNF-α and IFN-γ. Vitamin B_12_ assuaged the CaC_2_-induced elevation of these pro-inflammatory cytokines.

**Conclusions:**

These findings demonstrate for the first time that oral supplementation with vitamin B_12_ can protect mice against CaC_2_-mediated toxicity, inflammation and oxidative stress. The findings provide vital tools for forensic and diagnostic indicators for harmful CaC_2_ exposure; while providing useful insights into how vitamin B_12_ can be explored further as an adjunct therapy for CaC_2_ toxicity.

## Background

Calcium carbide (CaC_2_) is used in the manufacture of many compounds including acetylene gas. CaC_2_ yields acetylene gas when dissolved in water. Prior studies have demonstrated that when ingested by human, acetylene produces free radicals that causes cellular and organ damage [1, 2]**.** These CaC_2_—induced free radicals cause cellular damage and accelerate the aging process [3, 4]. In addition, it also contains impurities of arsenic and phosphorous compounds that are relatively toxic to human and animals [5, 6]. The growing awareness of fruit safety in regard to chemical exposure has awakened research about hazards in regard to contamination with CaC_2_ and a repertoire of other chemicals used in fruit ripening process [7, 8]. Furthermore, CaC_2_ is inappropriately used to chemically induce fruit ripening in many countries [9]. CaC_2_, is cheap and readily available tempting farmers to harvest their fruits before maturation. Even though artificial ripeners quicken the rate of the ripening process, the nutritional quality in regard to sensory, and safety of the fruits is compromised [2]. Evidently, CaC_2_ causes food contamination, gastric irritation, mouth ulcers, cerebral oedema, seizures, and changes in vital hematological and biochemical processes [2, 10]. Further reports have shown that chemical substances used as artificial ripeners have negative effects on humans that include memory loss, cerebral oedema, prostate, changes in DNA and RNA [2]. It is well established that CaC_2_ impacts the sensory system by limiting oxygen supply to the brain, with potential consequence of neural damage. In addition, evidence shows involvement in lung failure, renal failure, dermal diseases and heart conditions [10, 11]. There is a need for detailed studies to elucidate and characterize the negative physiological and biochemical process affected by CaC_2_ in humans to aid in diagnosing exposure and development of pharmaceutical intervention. Previous investigations suggest involvement of oxidative stress and inflammation in CaC_2_-driven organ damage [4]. It therefore makes sense that a potent antioxidant and anti-inflammatory agent could mitigate CaC_2_ toxicity. Hence cyanocobalamin also known as vitamin B_12_, a potent antioxidant and anti-inflammatory molecules, usually taken as a supplement, was investigated to determine its potential to mitigate CaC_2_ toxicities. Vitamin B_12_ is vital in the synthesis of red blood cells, keeping nerve cells healthy and synthesis of genetic materials. The sources of vitamin B_12_ include meat, milk, fish, and shellfish [12]. Vitamin B_12_ is a methyl donor precursor, naturally occurring in the diet. Previous studies have demonstrated that when _combin_ed with folate, vitamin B_12_ down-regulated the pro inflammatory cytokines and low-grade systemic inflammation [13]. The current study sought to profile or characterize negative physiological and biochemical effects of CaC_2_ exposure in a mouse model. In addition; the study sought to establish the effectiveness of vitamin B_12_ in abrogating CaC_2_–induced negative effects.

## Results

### Effect of calcium carbide and vitamin B_12_ on body and organ weight

**Fig. 1:**
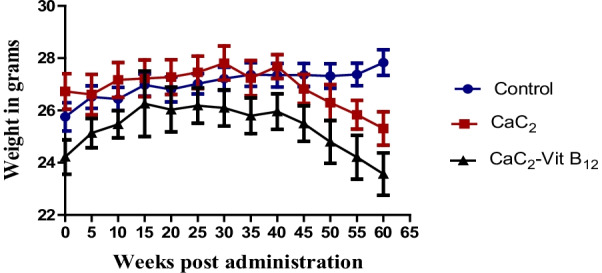
The effects of CaC_2_ and vitamin B_12_ on body weight. The change in body weight was analyzed by One-way ANOVA. n = 12. Bars presents ± SEM

**Fig. 2:**
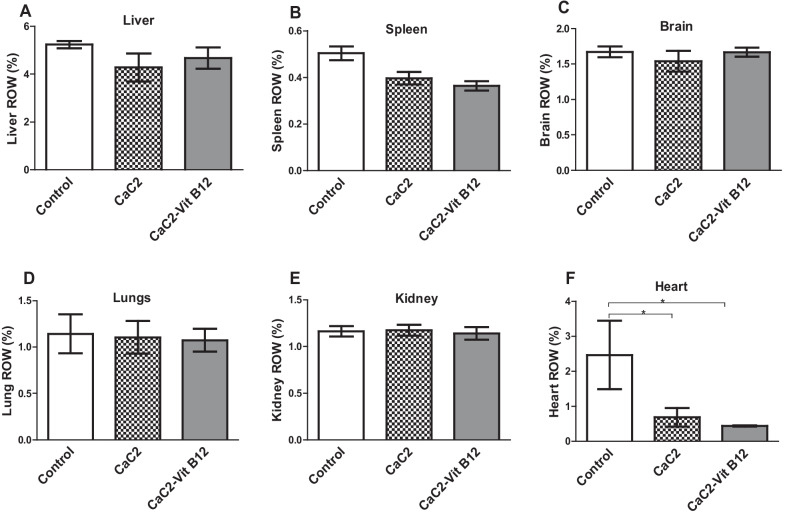
The effect of CaC_2_ and vitamin B_12_ on relative organ weight. Bar graphs showing change in organ weight for, Liver (**A**), Spleen (**B**), Brain (**C**), Lungs (**D**), Kidney (**E**) and Heart (**F**). The relative organ weight was analyzed by One-way ANOVA, followed by Tukeys Post hoc test (*P ≤ 0.05). n = 12. Bars presents ± SEM

This study examined whether vitamin B_12_ administration would restore CaC_2-_induced alteration in physiological parameters by measuring changes in general body and relative organ weight. Determination of mice body weight showed a steady increase in weight of control group of mice compared to the CaC_2_ supplemented group of mice (Fig. 1). There was a marginal weight gain in the group of mice both supplemented with CaC2 and/or with vitamin B12 up to 40 days post-administration; and decreased thereafter up to 60 days post-administration. To determine if the weight change was due to alteration in organ weights, the relative organ weights were determined. This was vital in order to determine whether the effect of oral administration of CaC_2_ potentiated organ injury and whether administration of vitamin B_12_ could ameliorate such injury. The relative organ weight for liver, spleen, brain, lungs and kidney were unaffected upon CaC_2_ exposure (Fig. 2A–E) and a significant decrease in the weight of the heart (Fig. 2F) in the CaC_2_-exposed groups. This results suggest that exposure to CaC_2_ has minimal impact on the general body weight.

### The effect of calcium carbide and vitamin B_12_ on packed cell volume (PCV), red blood cells (RBC) and haemoglobin (Hb)

**Fig. 3:**
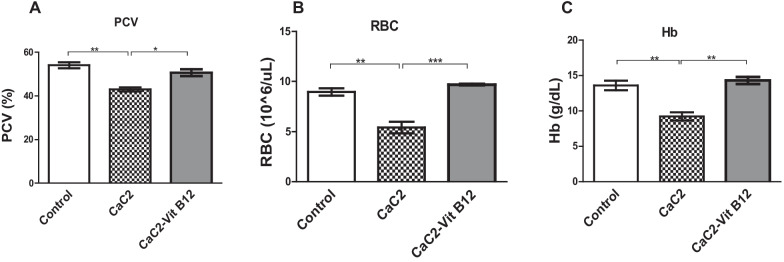
The effect of CaC_2_ and vitamin B_12_ on PCV, RBC and Hb. PCV (**A**), RBC (**B**) and Hb (**C**) levels were evaluated. Mean comparison was analyzed by One-way ANOVA, followed by Turkeys Post hoc test (*P ≤ 0.05; **P ≤ 0.01; ***P ≤ 0.001). n = 12. Bars presents ± SEM

This study aimed to investigate the modulating effect of vitamin B12 on PCV, RBC and Hb following CaC_2_ exposure. The altered blood levels of PCV, RBC and Hb were measured in the blood of CaC_2_ and vitamin B_12_ supplemented mice. The results showed that CaC_2_ significantly decreased (*P* < 0.0001) the levels of PCV (Fig. 3A). Notably, vitamin B_12_ when administered stabilized PCV levels. In the study, it was observed that CaC_2-_ significantly depleted the levels of RBCs (Fig. 3B) with concomitant decrease in the levels of Hb (Fig. 3C); a clear indication of anaemia. These results suggest that, administration of vitamin B_12_ protected mice from CaC_2_-induced anemia is linked to its anti-oxidant properties.

### Effect of calcium carbide and vitamin B_12_ on red blood cell (RBC) indices

**Fig. 4:**
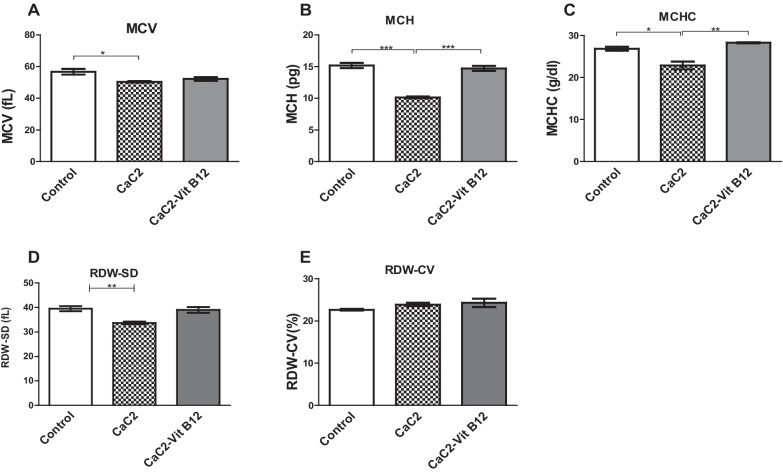
The effect of CaC_2_ and vitamin B_12_ on MCV (**A**), MCH (**B**), MCHC (**C**), RDW-SD (**D**) and RDW-CV (**E**). Mean comparison was analyzed by One-way ANOVA, followed by Turkeys Post hoc test (*P ≤ 0.05; **P ≤ 0.01; ***P ≤ 0.001). n = 12. Bars presents ± SEM

Next, this study sought to classify the type of anemia that was induced following CaC_2_ exposure. Classification was done by measuring various RBC indices in the blood. There was decrease in MCV levels upon exposure to CaC_2_ relative to control group of mice (Fig. 4A). Notably, vitamin B_12_ supplementation restored the significant CaC_2_-driven down-regulation of the mean corpuscular hemoglobin (MHC), the mean corpuscular hemoglobin concentration (MCHC) and ded cell distribution width standard deviation (RDW-SD) (Fig. 4B–D). However, the levels of red cell distribution width coefficient of variation (RDW-CV) (Fig. 4E) were comparable across all the groups. This result demonstrate the effect of protecting against CaC_2-_induced microcytic hypochromic anemia may be linked to the antioxidant impact of vitamin B_12_.

### Effect of calcium carbide and vitamin B_12_ on platelets (PLT)

**Fig. 5:**
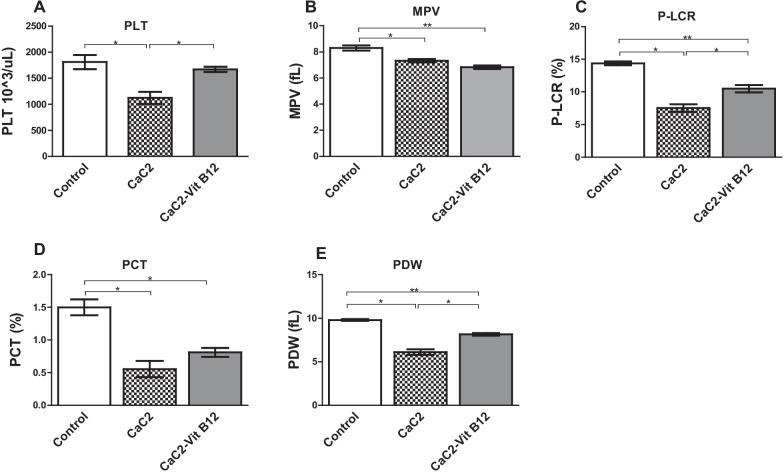
The effect of CaC_2_ and vitamin B_12_ on levels of platelets and platelet indices. The histogram shows levels of PLT (**A**), MPV (**B**), P-LCR (**C**), PCT (**D**) and PDW (**E**). Mean comparison of PLT and its sub-types were analyzed by One-way ANOVA, followed by Turkeys Post hoc test (*P ≤ 0.05; **P ≤ 0.01; ***P ≤ 0.001). n = 12. Bars presents ± SEM

The levels of PLT and its indices were measured in the blood of CaC_2_ exposed mice to determine if supplementation with vitamin B_12_ can reverse any alteration that may be linked to thrombocytopenia. CaC_2_-induced thrombocytopenia was evident through suppression of platelet levels. Intriguingly, vitamin B_12_ administration appeared to aid the recovery of platelet levels (Fig. 5A). Further analysis of the platelet indices showed that mean platelet volume (MPV) levels (Fig. 5B) in mice exposed to calcium carbide was depleted when compared to the normal control. The platelet large cell ratio (P-LCR) was significantly reduced (*P* < 0.05) in mice receiving calcium carbide (Fig. 5C) when compared to either control or vitamin B_12_ supplemented group of mice. This was further validated by significant reduction in the plateletcrit (PCT) and the platelet distribution width (PDW) in comparison to the control group mice (*P* < 0.05; Fig. 5D, E). The findings from this study demonstrate that vitamin B_12_ aided the recovery of platelets in the presence of CaC_2_.

### Effect of calcium carbide and vitamin B_12_ on white blood cells (WBCs) and its subtypes

**Fig. 6:**
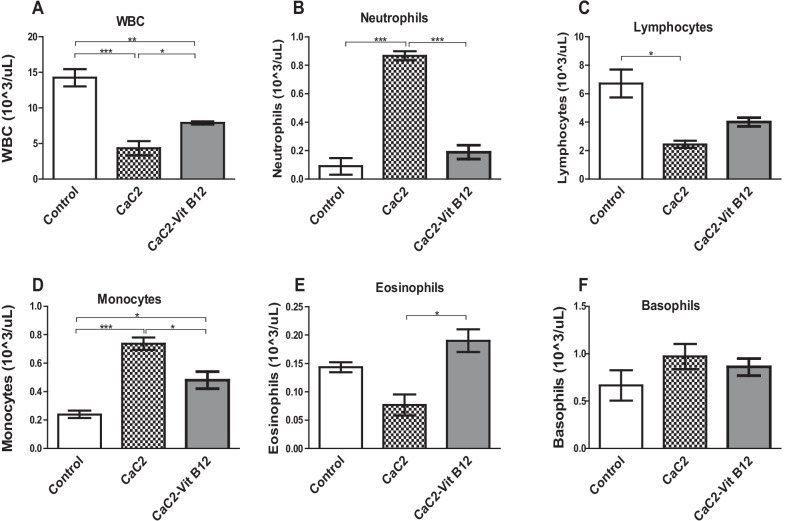
The effect of CaC_2_ and vitamin B_12_ on WBCs and various subtypes of WBCs. Mice were monitored for WBCs (**A**), Neutrophils (**B**), Lymphocytes (**C**), Monocytes (**D**), eosinophils (**E**) and Basophils (**F**). Mean comparison of WBCs and its sub-types were analyzed by One-way ANOVA, followed by Turkeys Post hoc test (*P ≤ 0.05; **P ≤ 0.01; ***P ≤ 0.001). n = 12. Bars presents ± SEM

An attempt to elucidate the putative effect of vitamin B_12_ to reverse the effects of CaC_2_-induced alteration of WBC was made. To address this question, the levels of WBC and WBC subtypes were measured in blood from the treatment group of mice. Normally, depletion of WBC numbers beyond a certain limit is an indication of leukopenia. Consistence with this, it was found out that the levels of WBCs were significantly decreased (*P* < 0.001) in mice administered with CaC_2_ compared to the control group (Fig. 6). Remarkably, exposure to vitamin B_12_ prevented CaC_2_-driven suppression of WBCs. Prior experiments have already shown that CaC_2_ has the capacity to induce leukopenia. Hence, this study sought to determine whether there was alteration in the composition of WBC phenotypes, in order to determine the extent of vulnerability of the mice to infections and signs of inflammation. Oral administration of CaC_2_ was found to significantly increase (*P* < 0.01) neutrophils with reduction in the lymphocytes (Fig. 6B, C respectively). However, exposure to CaC_2_ resulted in significant increase in monocyte levels with concomitant abrogation of eosinophil levels (Fig. 6D, E). Interestingly, vitamin B_12_ showed the ability to prevent the CaC_2_-induced elevation of monocytes and neutrophils and restored lymphocyte and eosinophils levels. On the other hand, the levels of basophils were comparable across all the treatment groups (Fig. 6F). These results suggests that vitamin B_12_ supplementation restored the CaC_2_-induced alteration in WBC and its subtypes.

### Effect of calcium carbide and vitamin B_12_ on the lipid profile

**Fig. 7:**
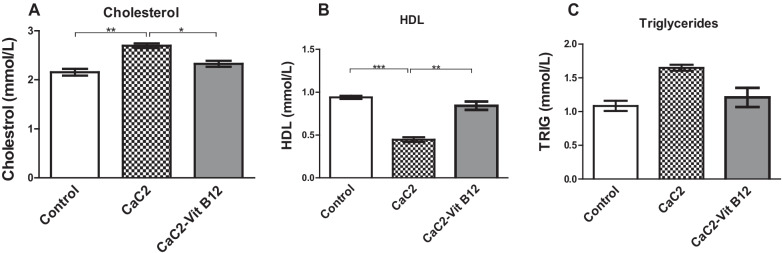
The effect of CaC_2_ and vitamin B_12_ on the lipid profile. Levels of total Cholesterol (**A**), HDL (**B**) and triglycerides (**C**) were evaluated. Mean comparison of lipid profile was analyzed by One-way ANOVA, followed by Turkeys Post hoc test (*P ≤ 0.05; **P ≤ 0.01; ***P ≤ 0.001). n = 12. Bars presents ± SEM

This study investigated whether vitamin B_12_ administration could restore the effect of CaC_2-_induced alteration of lipid profile by measuring the changes in the serum levels total cholesterol, high density lipoproteins and triglycerides. Results from this study further reveal that exposure of mice to CaC_2_ resulted in elevation of total cholesterol levels relative to either control or vitamin B_12_ administered mice (Fig. 7A). Notably, a significant decrease in high density lipoprotein (HDL) (*P* < 0.05) was observed in mice administered with CaC_2_ (Fig. 7B). On the contrary, the levels of triglycerides were comparable across all the treatment groups (Fig. 7C). This observation indicates that vitamin B_12_ was more effective in mitigating CaC_2-_induced elevation of cholesterol and reduction in high density lipoprotein, may be linked to the cellular protection.

### Effect of CaC_2_ and vitamin B_12_ on organ cellular reduced glutathione (GSH) concentration

**Fig. 8:**
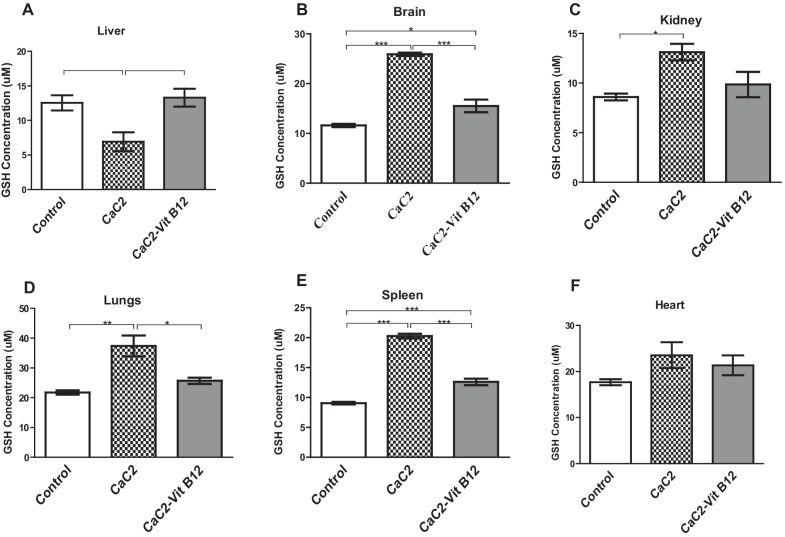
Effect of CaC_2_ and vitamin B_12_ on GSH levels in the liver (**A**), brain (**B**), kidney (**C**), lungs (**D**), spleen (**E**) and heart (**F**). Mean comparison of GSH levels was analyzed by One-way ANOVA, followed by Turkeys Post hoc test (*P ≤ 0.05; **P ≤ 0.01; ***P ≤ 0.001). n = 12. Bars presents ± SEM

Next we determined whether vitamin B_12_ supplementation had the capacity to modulate oxidative stress following exposure of mice with CaC_2_ by measuring the cellular levels of GSH in the liver, kidney, brain, spleen, lungs and heart. Reduced glutathione is one of the primary antioxidants involved in the quenching of reactive oxygen species (ROS) under abnormal physiological conditions. In the CaC_2_-treated group, there was significant decrease in the level of GSH in the liver relative to mice administered with vitamin B_12_ (Fig. 8A). Supplementation with vitamin B_12_ blocked suppression of the cellular GSH levels in the liver. Additionally, exposure of mice to CaC_2_ resulted in a significant increase (*P* < 0.05) in the concentration of cellular GSH levels in the brain, kidney, lungs and spleen (Fig. 8B–E). However, the cellular levels of in the heart were comparable across all the treatment groups (Fig. 8F). Vitamin B_12_ supplementation resulted in stabilization of GSH levels in this vital organs. These results is a clear indication of reduced oxidative stress in the presence of vitamin B_12_.

### Effects of calcium carbide and vitamin B_12_ on the markers of liver and kidney injury

**Fig. 9:**
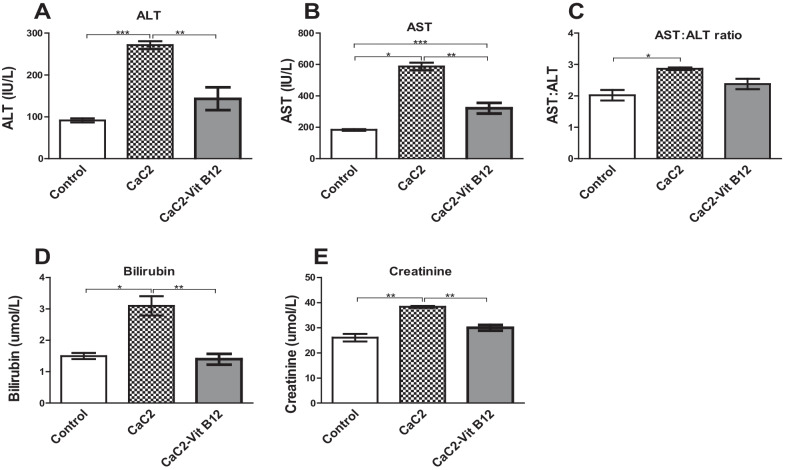
Effect of CaC_2_ and vitamin B_12_ on ALT (**A**), AST (**B**), and AST: ALT (**C**) ratio and total bilirubin (**D**) and creatinine (**E**). Female Swiss white mice were orally administered with calcium carbide and vitamin B_12_. Comparison among the treatment groups was analyzed by One-way ANOVA, followed by Turkeys Post hoc test (*P ≤ 0.05; **P ≤ 0.01; ***P ≤ 0.001). n = 12. Bars presents ± SEM

This study determined whether vitamin B_12_ supplementation would nullify CaC_2_-induced liver and kidney injury, by measuring serum levels of transaminases, total bilirubin and creatinine. Calcium carbide administration resulted in elevation of ALT and AST levels (Fig. 9A, B). Similarly, the result showed a ratio of more than 2:1 in regard to AST: ALT, indicative of liver damage (Fig. 9C). In the presence of vitamin B_12_, CaC_2_-driven rise in ALT and AST was blocked. Having clearly established the putative impact of oral administration of vitamin B_12_ on elevated on liver transaminases, in the presence of CaC_2,_ it was important to determine the extent of CaC_2_-induced livery injury through the measurement of bilirubin. It was observed that administration of vitamin B_12_ after CaC_2_ exposure was effective in restoring CaC_2-_induced elevation of levels of total bilirubin (Fig. 9D). There was a significant increase (*P* < 0.05) in levels of the serum creatinine among mice treated with CaC_2_. Oral administration of vitamin B_12_ significantly abrogated CaC_2-_induced elevation of serum creatinine levels (Fig. 9E). These results suggest that vitamin B_12_ supplementation protected against CaC_2-_induced liver and kidney injury is associated with anti-inflammatory properties of vitamin B_12_.

### Histopathological analysis of liver and kidney

**Fig. 10:**
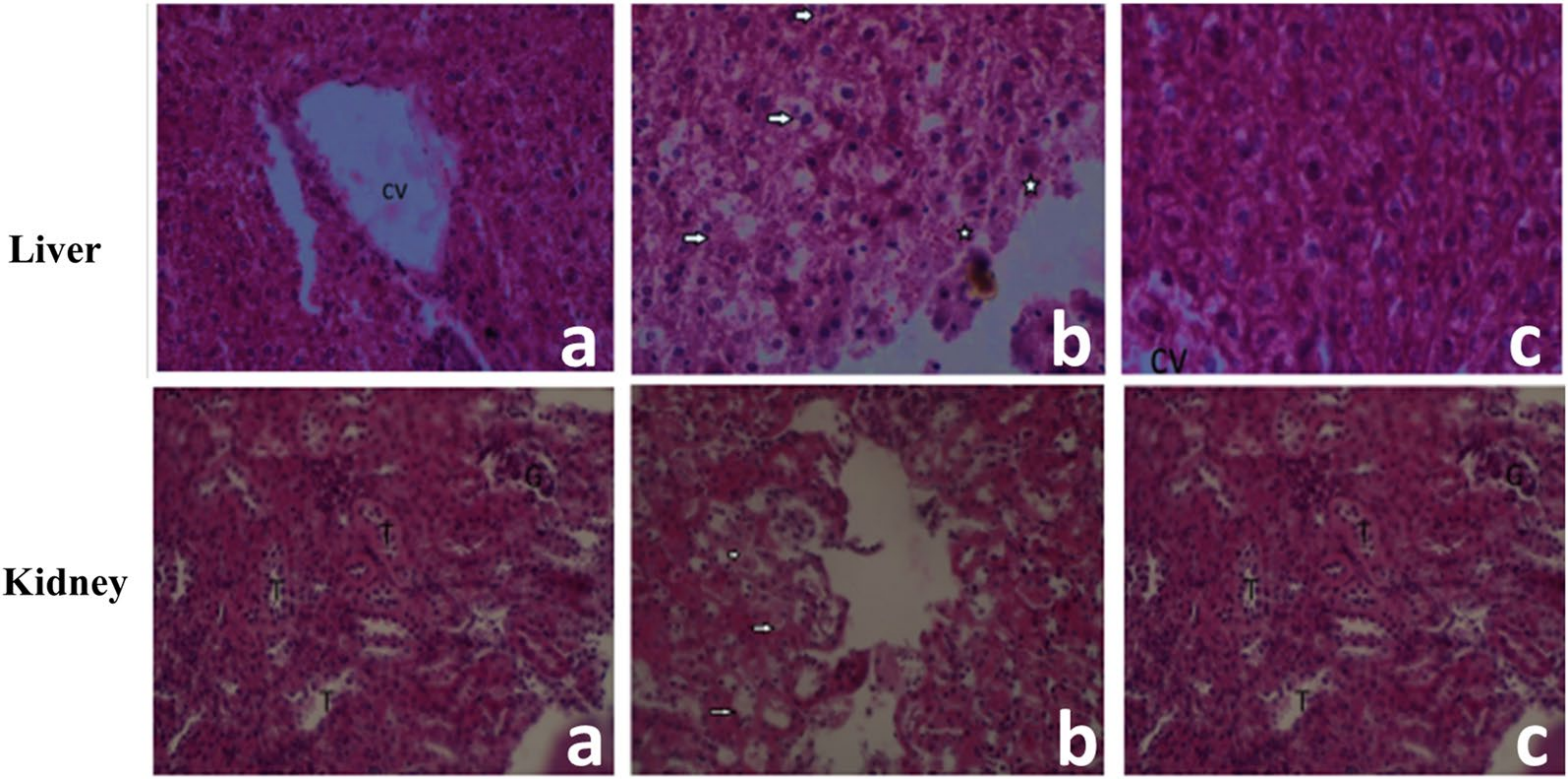
Effect of CaC_2_ and vitamin B_12_ on liver tissues from mice. Vitamin B_12_ was given to assess extent of protection against CaC_2_ exposure. Liver and kidney tissues from Control group (**a**), CaC_2_ group (**b**), and CaC_2_-Vitamin B_12_ group (**c**) were processed for histology with H&E staining. Images show representative liver sections with the hepatocyte necrosis (CV-Central vein, arrows indicated diffuse hepatocyte swelling and necrosis and stars are focal areas of hemorrhages in Liver parenchyma). From the kidney sections with the hepatocyte necrosis (G-Glomerulus; T-Renal tubules & Arrow- Cytoplasmic vacuolation of tubular epithelia cells). (Original magnification × 400)

To establish presence of any deleterious effects of CaC_2_ administration on the liver cyto-architecture, we performed normal standard histopathological analysis. Exposure to CaC_2_ resulted in significant damage to the liver was characterized by diffuse hepatocyte swelling necrosis, and focal hemorrhages in the liver parenchyma (Fig. 10). It is noteworthy, that Vitamin B_12_ administration CaC_2_ assuaged CaC_2_-driven liver pathology. Furthermore, CaC_2_ administration resulted in kidney damage. Specifically, the injury was characterized by the presence of cytoplasmic vacuolation of tubular epithelial cells (Fig. 10). These results suggest that administration of vitamin B_12_ protected liver and kidney tissue from CaC_2_ -induced damage is associated with the anti-inflammatory properties of vitamin B_12_.

### Effect of calcium carbide and vitamin B_12_ on cytokines

**Fig. 11:**
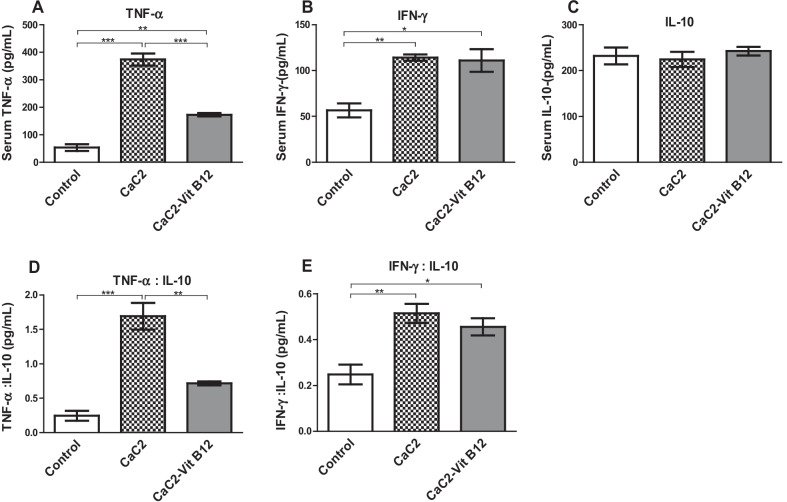
Effect of CaC_2_ and vitamin B_12_ on pro -inflammation cytokines. Female Swiss white mice were orally administered with calcium carbide and vitamin B_12_. TNF-α (**A**), IFN-γ (**B**), and IL-10 (**C**), TNF-α: IL-10 (**D**) and IFN-γ: IL-10 (**E**) ratios were evaluated. Comparison of serum cytokine levels among the treatment groups was analyzed by One-way ANOVA, followed by Turkeys Post hoc test (*P ≤ 0.05; **P ≤ 0.01; ***P ≤ 0.001). n = 12. Bars presents ± SEM

The levels of the pro-inflammatory cytokines tumor necrotic factor-alpha (TNF-α) and interferon gamma (IFN-γ), as well as the anti-inflammatory cytokine interleukin-10 (IL-10), were determined from serum samples to evaluate the ability of CaC_2_ to trigger inflammation. CaC_2_ markedly elevated levels of serum TNF-α and IFN-γ (*P* < 0.0001) (Fig. 11A, B). However, the levels of serum IL-10 were similar across the treatment groups (Fig. 11C). In the presence vitamin B_12,_ the elevation of serum TNF-α and IFN-γ levels was abrogated. The ratios between pro-inflammatory and anti- inflammatory cytokines established the extent of active inflammation where CaC_2_ supplementation resulted in significant (*P* < 0.05) imbalance of both TNF-α: IL-10 ratio and IFN-γ: IL-10 ratios (Fig. 11D, E). However, vitamin B_12_ supplementation abrogated the CaC_2_-induced TNF-α: IL-10 imbalance. These results demonstrates the effect of protecting against CaC_2-_induced inflammation can be linked to the anti-inflammatory effect of vitamin B_12_.

## Discussion

Exposure to CaC_2_ causes serious detrimental effects that often can trigger cancer development, food poisoning, irritation of gastral tissues and mouth ulceration, cerebral oedema and seizures [2]. Moreover, ingestion of fruits ripened with CaC_2_ can cause alterations to vital physiological and biochemical processes [7]. Notably, the negative physiological and biochemical processes due to CaC_2_ exposure have not been well characterized to enable diagnostic and forensic determination of exposure, as well as allow development of detoxification or treatment strategies. In this study, we demonstrate for the first time that vitamin B_12_, a potent anti-inflammatory and antioxidant, assuaged CaC_2_-induced negative physiological and biochemical effects in a mouse model. Moreover, the findings from the present study established that exposure of mice to only vitamin B_12_ alone did not have any effect on both physiological, biochemical and inflammatory responses (data not provided). Notably, oral exposure to CaC_2_ resulted in marginal decrease in mice body weight, though not statistically significant. Other studies have reported significant CaC_2_–induced weight gain in wistar rats [7]. The marginal alteration in body weight due to CaC_2_ relative to the control indicates interference with normal physiological growth and development; which can be attributed to the impairment of biochemical processes critical for normal growth and loss of appetite. Notably, administration of vitamin B_12_ did not have any impact on the general body weight of mice due to CaC_2_ exposure, or that of its organs, probably due to its contribution in inhibition of adipocyte differentiation and lipid accumulation. Worthy of note is that the exposure to CaC_2_ resulted in significant decrease in the relative organ weight of the heart; which was not rescued by vitamin B12 supplementation. Indeed, increased levels of AST in the current study further shows the possibility of CaC_2_-induced toxicity in other organs such as the heart; given that AST is ubiquitously distributed in other major organs of the body. Recently it has been reported that cardiovascular dysfunction due to CaC2 is associated with inflammatory mediators and oxidative stress [14]. Indeed, this findings imply that exposure to CaC_2_ can result in a high risk of heart diseases This is a significant and profound phenomenon that warrants further investigations especially on the histopathological analysis of the heart.

Hematopoiesis is a very significant process that warranties effective and controlled supply of a range of blood cellular constituents [15]. This study reports clear evidence for CaC_2_-driven derangement of hematopoiesis. Reduction in PCV has been associated with oxidative stress and impaired osmoregulation [8]. In the current study, a significant CaC_2_-driven decrease in PCV was noted. Decrease in PCV may be attributed to harmful effect of calcium carbide on the bone marrow, alteration of micronutrients for RBC synthesis, inhibition of erythropoietin and elevated haemolysis of the red cells due to noxious consequence. A similar study in which mice were fed on fruits ripened by CaC_2_ showed reduced PCV levels [11]. Indeed, a decline in PCV is one of the key features of exposure to toxic chemicals that is associated with generation of ROS and lipid peroxidation [16]. Notably, administration of vitamin B_12_ significantly stabilized the PCV levels which may be attributed to its anti-inflammatory properties. Vitamin B_12_ scavenges free radicals and enhances membrane integrity by preventing lipid oxidation [17]. Indeed, lipid oxidation is interrelated with membrane fragmentation and ultimate cell death, then it is possible that vitamin B_12_ protected against CaC2-driven decline in PCV levels by quenching levels of free radicals in blood thus protecting the red blood cells membrane against oxidative damage.

There was a clear CaC_2_-induced reduction in the levels of the RBCs and hemoglobin; indicative of anemia. The present findings are consistent with prior study in which expose to CaC_2_ resulted in significant decrease in Red Blood Cell, Haemoglobin and Packed Cell Volume count [7]. CaC_2_ driven decrease in RBCs and Hb may be due to several factors including impaired erythropoiesis, accelerated RBC lysis and microcytic or normocytic anaemia [1]. Notably, CaC_2_ has been shown to obstruct elements and minerals important for erythropoiesis such as iron, folic acid and vitamin B_12_ [2]. It is noteworthy that decreased haemoglobin content may result from lyses of erythrocytes, erythropenia, haemopoiesis and hindrance of erythropoietin or disruption of haemoglobin production [1]. Previous studies have shown that vitamin B_12_ administration was more effective in stimulating erythropoiesis among premature infants [18]. A remarkable finding here was that administration of vitamin B_12_ protected mice from CaC_2-_ induced suppression of RBCs and Hb levels. This results strongly supports the ameliorative effect of vitamin B_12_ in CaC_2_-induced anemia observed in the current study.

Changes in RBC indices (MCV, MCH, and MCHC) constitutes a key parameter applied in the classification of anaemia. Herein, it was observed that CaC_2_-induced microcytic hypochromic anaemia, as shown by the significant reduction in the levels of MCH, MCHC and RDW-SD. Note that, contrary findings have been reported in CaC_2_ exposure to rats; where RBC indices were up-regulated [7]. Once again, we demonstrated that vitamin B_12_ supplementation significantly restored the levels of red cell indices. This is not surprising given that vitamin B_12_ is vital for DNA synthesis and its deficiency can cause megaloblastic anaemia via abortive erythropoiesis and hyperbilirubinemias.

Platelets (PLT) play a critical role in blood clotting cascades, and are vital for preventing bleeding, support healing and have also been involved in inflammatory response and wound healing [19]. In the current study, CaC_2_ significantly suppressed platelet levels, possibly through the initiation of caspase-dependent apoptosis. Reduction in platelet count may also be an indication of thrombocytopenia, whose cause may be impaired hematopoiesis; which may constitute a serious health threat to people on blood thinning therapy [20, 21]. Vitamin B_12_ administration appeared to aid the recovery of platelet levels. Notably, vitamin B_12_ seems to stimulate thrombocytosis, possibly due to anti-inflammatory effects on vital molecules crucial for regulating platelet levels and modulate blood clotting cascades. This phenomena warrants further scrutiny.

White blood cells (WBCs) play an important role in immune function [10]. In the current study, a significant decrease in WBCs was recorded in mice administered with CaC_2_. Decrease in the WBCs also referred to as leukopenia due to CaC_2_ portends serious consequences in regard to the ability to fight infections as well as disease diagnostic tests that rely on WBC levels. Leukopenia in mice administered with CaC_2_ is indicative of CaC_2_-induced severe suppression of lymphoproliferative processes. Similar findings have been reported in other related studies [11]. Notably, other studies have shown contradictory findings in rats fed on fruits ripened with CaC_2_ [10]. Further, studies established the ability of vitamin B_12_ to prevent CaC_2_-driven down-regulation of WBCs when administered. We further evaluated the effect of CaC_2_ on the various WBC subtypes (lymphocytes, monocytes, basophil and neutrophils). Oral administration of CaC_2_ significantly increased the levels of neutrophils and resulted in reduced lymphocyte levels. CaC_2_-driven neutrophilia and monocytosis was noted. Notably, basophil levels were unchanged. However, in the presence of vitamin B_12_, lymphocyte levels were stabilized in mice administered with CaC_2_. Neutrophilia could be triggered due to CaC_2_-driven stress as well as inflammation and cellular damage. Neutrophilia, monocytosis and suppression of lymphocytes would definitely have detrimental implications in disease diagnosis. For example, elevation of neutrophils (neutrophilia) is characterized by laectanae infection. On the other hand, high lymphocytes and monocytes (monocytosis) traditionally signal an infection and could signal a serious disease such as leukemia. Moreover, eosinophilic is an indication for a parasitic infection, allergic reactions or cancer. The profound conclusion based on our findings is that CaC_2_-driven neutrophilia, monocytosis and suppression of lymphocytes can interfere with critical laboratory diagnostic data for serious bacterial or viral infections as well as diseases like cancer. Certainly, this phenomenon requires further scrutiny. Additionally, it is necessary to investigate this possibility in the future in order to determine the underlying mechanism through which CaC_2_ mediate the derangement of hematopoiesis process within the bone marrow.

Further investigations sought to elucidate the impact of CaC_2_ and vitamin B_12_ on lipid metabolism. Exposure of mice to CaC_2_, resulted in significant elevation of cholesterol. Notably, a significant decrease in high density lipoprotein (HDL) was observed in mice administered with CaC_2_. On the other hand, the levels of triglycerides were comparable across the groups. Previous studies have noted contradictory findings in regard to the effect of CaC_2_ on lipid metabolism. A previous study reported decreased plasma cholesterol and low-density lipoprotein (LDL) in rats fed on mangoes ripened with CaC_2_ [7]. Note that HDL stimulates efflux of excessive cholesterol from outlying tissues by reverting it back to the liver for biliary elimination [22]. Elevated serum levels of cholesterol, triglycerides (TG) and low density lipoprotein (LDL) is associated with dyslipidemia [23]. In pre-clinical studies, low vitamin B_12_ levels is linked to increased lipid accumulation in adipocytes that ultimately elicit dyslipidemia in mice [24]. On this basis, it can be concluded that CaC_2_ significantly affect lipid metabolism, with exposure to vitamin B_12_ counteracting this effect.

Glutathione (GSH) is a major antioxidant defense molecule produced in the body [25]. It is one of the most critical sources of reducing power and redox stabilization in cells [26]. Consequently, it provides first line cellular defense against free radicals. Reduced glutathione in the tissues is one of the primary antioxidants involved in the quenching of generated ROS under abnormal physiological conditions such as those induced by toxins [27, 28]. Hydroxamic acid, a known toxin, disrupts the antioxidant balance in the liver and spleen at higher doses in rats [27]. For this reason, changes in cellular GSH can be utilized as a marker for oxidative stress [29]. In the current study, a significant depletion of GSH was noted in the liver. Reduced levels of GSH in body tissues is suggestive of extreme oxidative stress. Calcium carbide administration resulted in a significant increase in the concentration of cellular GSH levels in the brain, kidney and lungs. However, the cellular levels of GSH in the spleen and heart were comparable across all the treatment groups. A rise in GSH levels may be due to induction of its synthesis via a cascade of enzymatic reactions; in response to rise in oxidative stress [26, 29]. On the other hand, decreased GSH may be caused by its overutilization during chronic oxidative stress due to its role as the prime intra cellular anti-oxidant [26]. Vitamin B_12_ supplementation resulted in stabilization of GSH in the presence of CaC_2_ in the liver, brain and kidney. This is not surprising given that Vitamin B_12_ is known to prevent deleterious effects associated with oxidative tissue injury due to its anti-apoptotic and anti-oxidative functions. It is noteworthy, to mention that other previous studies have demonstrated the role of vitamin B_12_ in stabilizing GSH levels [27, 30]. This may attribute to the ability of vitamin B_12_ to counter oxidative stress, thereby reducing the demand on GSH due to its anti-oxidant properties.

The liver is an important organ that plays a vital role in the metabolism of xenobiotics and is the primary target for CaC_2_ toxicity. Therefore, during CaC2 poisoning, liver damage is unavoidable.

Moreover, ALT and AST are important liver enzymes, whose levels in serum is an indicator of liver pathology. In the current study, ALT and AST levels were elevated upon exposure of mice to CaC_2_, but were stabilized upon administration of vitamin B_12_. Additional investigations on the liver showed CaC_2_-driven elevation of bilirubin. A bilirubin test is vital in diagnosing liver damage since bilirubin is a bile pigment formed from the breakdown of haemoglobin in RBCs. Increased bilirubin levels is indicative of hepatobiliary disorder with blockage of flow of bile through the bile duct [7]. Perhaps due to its antioxidant capabilities, vitamin B_12_ stabilized levels of AST, ALT and bilirubin. This a clear evidence for Vitamin B_12_-driven hepatocellular protection from CaC_2_-driven liver injury [31].

Additional investigations focused on the integrity of kidney function in the presence of CaC_2_ and vitamin B_12_. We demonstrate that CaC_2_ driven renal injury was ameliorated by administration of vitamin B_12_. Elevated creatinine levels suggest a reduction in the glomerular filtration rate; indicative of kidney function impairment. Similar findings have been noted in rat fed with CaC_2_-ripened mango [1]. Moreover, elevated creatinine levels is associated with reduced glomerular filtration, impaired elimination of waste products with potential kidney swelling, inflammation, and necrotic cell damage [16]. Once again, oral administration of vitamin B_12_ abrogated CaC_2-_induced rise in serum creatinine levels is linked to its anti-inflammatory properties.

Cytokines are biologically active proteins that mediate vital intercellular communication in the immune system and are secreted by different immune cell types. In addition, they participate in host defense, inflammatory and tissue repair activities [32, 33]. Pro-inflammatory cytokines are produced by activated macrophages effector cells that participated in adaptive immune system and play a significant role in exacerbation of inflammatory processes. Consequently, interferon gamma (IFN-γ) and tumor necrosis factor alpha (TNF-α) are critical in fighting infections, and ultimately for cell survival mechanisms. In the current study, the levels of the pro-inflammatory cytokines tumor necrotic factor-alpha (TNF-α) and interferon gamma (IFN-γ), as well as the anti-inflammatory cytokine interleukin-10 (IL-10), were determined from serum samples to evaluate the ability of CaC_2_ to trigger inflammation. CaC_2_ supplementation resulted in markedly augmented levels of serum TNF-α and IFN-γ. Ordinarily, cytokines work in synergy with IFN-γ, and stimulates migration of immune cells to infection sites, leading to granuloma development, capable of regulating the immune response. The functions of IFN-γ in macrophage activation and stimulation of the antigen presentation cascades is well established [33, 34]. In the presence of vitamin B_12_, CaC_2_-driven elevation of serum TNF-α was blocked.

Anti-inflammatory cytokines such as interleukin 10 (IL-10), are immunoregulatory molecules that regulate the pro-inflammatory cytokine reaction. IL-10 is produced by leukocytes and is associated with inflammatory and autoimmune responses [35]. Furthermore, IL-10 exerts anti-inflammatory properties by inhibiting transcription factor; consequently targeting antigen-presenting cells and lymphocytes [32, 36]. Our findings demonstrated a CaC_2_-induced elevation of the pro-inflammatory cytokines, indicative of inflammation.

Vitamin B_12_ play a critical and vital role in the proper function of immune system chiefly as an immune-modulator [37]. Specifically, it has been observed that individuals who are deficient of Vitamin B_12_ have low levels of CD8+ T cells and impaired activity of NK cells [38]. Indeed, lambs put on vitamin B_12_ deficient diet were found to suffer most from *Mycobacterium paratuberculosis due to low lymphoblastic proliferation response further highlighting the significance of vitamin B*_*12*_* as an immune-stimulator* [39]. Moreover, vitamin B_12_ has been reported to favor both humoral and cellular immunity by increasing the levels of serum IgG, IgA, and IgM [38].

It is worth noting that vitamin B_12_ may have had an immunomodulatory effect that attenuated CaC_2_ toxicity driven by inflammation. Moreover, Vitamin B_12_ reduces homocysteine levels and inflammation hence regulating production of TNF-alpha. Note that vitamin B_12_ has also been adversely linked to pro-inflammatory cytokines and low-grade systemic inflammation in some studies [13].

Abnormal and continuous secretion with concomitant accumulation of ROS during CaC_2_ metabolism is linked with aggravation in organ toxicities. This study further investigated the effect of CaC_2_ through histopathological analysis of the liver and kidney to support biochemical analysis data. Exposure to CaC_2_ resulted in significant damage to the liver. The liver injury was characterized by diffuse hepatocyte swelling, necrosis, and focal hemorrhages in liver parenchyma. The cytoplasm of the hepatocytes appeared normal with basophilic nucleus. Hepatocytes appeared apoptotic and shrunken. Some of the degenerating cells were shrunken and looked like minute, structure-less, hyaline masses. Vitamin B_12_ administration exposure assuaged the CaC_2_ -induced tissue and hepato-cellular damage.

The kidney is an essential organ controlling vital physiological and biochemical processes such as homeostasis, detoxification and elimination of lethal metabolites and drugs [40]. Histopathological analysis of the kidney revealed evidence for CaC_2 -_induced kidney injury, characterized by the presence of cytoplasmic vacuolation of tubular epithelial cells. It can only be concluded that kidney injury and nephrosis occurred due to calcium carbide and/or its metabolites. The findings are consistent with studies conducted on proliferative and nonproliferative lesions of the rat and mouse urinary systems due to calcium carbide on rat tissues [4]. It was notable that administration of vitamin B_12_ after CaC_2_ protected kidney tissue from CaC_2_-induced damage. The association between vitamin B_12_ and reduced inflammation and homocysteine which is associated with oxidative stress has widely been reported by a number of studies [41, 42]. The capacity of vitamin B12 to be well distributed in extracellular and cytosolic spaces is fundamental in its efficiency against CaC_2_ toxicity. Further research will be needed to further characterize CaC_2_-toxicity in order to elucidate the apparent molecular processes responsible for the ability of vitamin B_12_ to confer protection from CaC_2_ toxicity in mice and further determine the residue levels of CaC_2_ toxicity. Moreover, there is need for future studies to look at the impact of CaC_2_ exposure on male mice and compare the findings from studies on female mice to see whether there exist any apparent differences in terms of sex.

## Conclusions

In this study we demonstrated a clear pattern of CaC_2_-induced interference with hematopoiesis, major organ function (liver, kidney), immune function, and oxidation status. A clear protective effect against these CaC_2_-driven assault by cyanocobalamin has been established. With further studies, a mitigation strategy against CaC_2_ toxicity using cyanocobalamin holds great promise.

## Methods

### Ethical statement

Experimental procedures and protocols involving use of mice adhered to International standards on laboratory animal use with strict adherence of the 3R rules and the ARRIVE checklist for handling animal research. The ethical clearance governing the use of mice in this study was approved by Institutional review for approval Committee (IRC) of the Institute of Primate Research Karen, Kenya (ISERC/08/2017).

### Experimental animals

In this study 5–6-week-old female Swiss white mice were purchased were from Biotechnology Research Institute Muguga, Kenya and were left to acclimatize for one week before the start of the experiment. Mice were housed in standard clean cages under a controlled room temperature of 21–25 °C and a 12 h light/dark cycle. Mice had access to clean water ad libitum and mice were fed on standard chow diet (Unga Group Plc., Nairobi, Kenya).

### Experimental design

Mice were randomly allocated into three treatment groups (n = 12). The treatments were administered orally at a dose of (100 mg/kg) Calcium carbide and (6 mg/kg) vitamin B_12_ all purchased from Sigma-Aldrich (Sigma-Aldrich Co., St. Louis, MO, USA). The calcium carbide sub-acute dosage was informed by previous findings that 500 mg/kg induced damage to vital organs [43]**.** A dose of 6 mg/kg was used for Vitamin B_12_ treatment in this study based on previous findings that 6 mg/kg enhance protection against xenobiotic induced toxicity [44]. Control group of mice received distilled water (vehicle), group two received calcium carbide dissolved in distilled water daily for 60 days, group three was administered with vitamin B_12_ after exposure to calcium carbide daily for 60 days. Mice were sacrificed at 60 days’ post treatment.

### Sample collection

Mice from each group were sacrificed as per the experimental design (60 days post treatment) by administration of ketamine (50 mg/ml) and xylazine (100 mg/ml) (Merck KGaA., Darmstadt, Germany) in a ratio of 4:1 through intramuscular injection to euthanize the mice. Anaesthetized mice were intracardially perfused with sterile phosphate buffer solution (PBS) to clear both non-adhering and adhering blood lymphocytes and erythrocytes. Brain, lungs, heart, kidney, liver and spleen samples were extracted and placed in 1.5 µl eppendorf tubes and collagenase (Sigma-Aldrich Co., St. Louis, MO, USA).

### Determination of body and organs weights

Change in general body weight was determined daily throughout the experimental period. After 60 days, mice from each group were anesthetized with 0.02 ml Ketamine and dissected to obtain liver, brain, lungs, heart, kidney and spleen. Analytical electronic balance (Mettler PM34, DoltaRange®., Mumbai, India) was used to measure the weight of the extracted organs. Individual relative organ weight were determined by dividing each animal’s organ weight by their body weight multiplied by 100%.

### Determination of haematological indices and biochemical markers

For hematological analysis, blood samples were obtained intra-cardially and were placed in EDTA tubes while blood for biochemical assay was collected in non-heparinized tubes and then they were centrifuged to obtain serum. Full blood hemogram was analyzed using automated Benchman Coulter counter (Benchman, Indianapolis, USA). Blood was left to stand at room temperature then centrifuged at 10,000 rpm for 5 min at 4 °C. Serum obtained was used to measure the levels of aspartate amino transferase (AST), alanine amino transferase (ALT), total bilirubin (TBIL) and creatinine were measured using automated analyzer (COBAS Integra-400 plus analyzer, Basel, Switzerland).

### Glutathione (GSH) assay determination

Snap frozen whole kidney, spleen, lungs, heart, brain and liver were homogenized using ice water at (4 °C) in 0.5 ml of 0.25 M sucrose, 5 mM Hepes-Tris, pH 7.5, with protease inhibitor cocktail whose concentration was 100% (w/v). Cellular levels of GSH (Sigma-Aldrich Co., St. Louis, MO, USA) was determined by employing the method of Griffith [45]. Briefly the cellular GSH from various organs was assessed by mixing the organ homogenates 5.5′ Dithiobis-2-nitrobenzoic acid (DNTB), with the absorbance of the resulting reaction product measured at 412 nm using a multi-detection microtitre plate reader (Thermo Fisher Scientific Inc., Wilmington, MA, USA).

### Enzyme linked immunoassay (ELISA)

ELISA secreted cytokine levels in serum for TNF-α, IFN-γ and IL-10 EISA kits (Thermo Fisher Scientific Inc., California, USA) were employed according to the manufacturer’s protocols. Briefly, High binding ELISA plates were coated with capture antibody and incubated at 4 °C overnight and washed 3 times using washing buffer followed by blocking using ELISA diluent and incubated for 1 h at room temperature. The plates were then washed followed by additional of standard cytokines and samples to the appropriate wells. The plates were then incubated for 2 h at room temperature, and then washed after which detection antibody was added and then incubated for 1 h at room temperature. The plates were then washed and then secondary antibodies were added and the plates were incubated for 1 h, after which they were washed and the substrate was added and incubated for 15 min at room temperature. Serum levels of these cytokine were quantified by ELISA micro-titer reader (Thermo Fisher Scientific Inc., Wilmington, MA, USA) at absorbance of 450 nm.

### Standard histopathology for the liver and kidney

Liver and kidney were harvested and rinsed in phosphate buffered saline and then fixed in 4% formaldehyde. Processing was done by dehydration at different concentration of alcohol and embedding them in paraffin wax. The tissues were then sectioned in thickness of 5 μm using HM 310 rotary microtome followed by staining of sectioned tissues with haematoxylin and eosin (H&E, Sigma-Aldrich Co.). The tissues sections were then analyzed by employing the use of compound microscope for pathological lesions.

### Statistical analysis

One-way ANOVA was used to compare the treatment groups with controls. For internal comparisons, Turkey’s post-hoc test was used. The results were given as a ± SEM with significance set at *P* < 0.05. Statistical analysis was done using GraphPad prism software package (Version 5.0).

## Data Availability

All the data that support the findings of this study are available on request from the corresponding author.

## Abbreviations

CaC_2_: Calcium carbide
PCV: Packed cell volume
Hb: Hemoglobin
RBC: Red blood cells
AST: Aspartate aminotransferase
ALT: Alanine aminotransferase
GSH: Reduced glutathione
ROS: Reactive oxygen species
TNF-α: Tumor necrotic factor-alpha
IFN-γ: Interferon gamma
